# The influence of calcium supplement on body composition, weight loss and insulin resistance in obese adults receiving low calorie diet

**Published:** 2010

**Authors:** Maryam Shalileh, Farzad Shidfar, Hamid Haghani, Shahriar Eghtesadi, Iraj Heydari

**Affiliations:** a Nutrition Sciences Department, Faculty of Health, Iran University of Medical Sciences, Tehran, Iran; b Statistics Department, Faculty of Midwifery, Iran University of Medical Sciences, Tehran, Iran; c Endocrinology Department, Faculty of Medicine, Iran University of Medical Sciences, Tehran, Iran

**Keywords:** Calcium Supplement, Insulin Resistance, Body Composition, Obesity, Weight Loss

## Abstract

**BACKGROUND:**

Obesity and diabetes are the most important problems of public health. Evidence from molecular animal research and epidemiologic investigations indicate that calcium intake may have an influence on body composition, weight and insulin resistance. The objective of this study was to determine the effects of calcium supplementation on body composition, weight, insulin resistance and blood pressure in the face of calorie restriction in obese adults.

**METHODS:**

A double blind randomized placebo-controlled trial on 40 adults with Body Mass Index > 25kg/m^2^ was conducted. Subjects were maintained for 24 weeks on a balanced deficit diet (-500 kcal/d deficit) and randomly assigned into two groups with 1000 mg ca/d as calcium carbonate or placebo.

**RESULTS:**

There were no significant differences in variables at the 12th and 24th week between the two groups. The lean mass showed no significant increase in the calcium group at the 12th week compared to baseline and in placebo group at the 24th week compared to the 12th week. The insulin concentration showed a significant decrease in the calcium group at the 12th week compared to the baseline (p < 0.05). The diastolic blood pressure had a significant decrease at the 24th week compared to the 12th week in both groups (p = 0.013-0.009).

**CONCLUSIONS:**

Results from this study suggest that 24 weeks of supplementation with 1000 mg ca/d did not have any effect on weight, body composition, insulin resistance and blood pressure beyond what can be achieved in an energy restricted diet in obese adults.

According to the WHO’s projections in 2005 nearly 1.6 billion adults were overweight and at least 400 million adults were obese in world.^1^ While obesity is related to multiple disease outcomes,^2^ it is also a risk factor for certain chronic diseases,^3^ such as diabetes.^1^

Diet has been reported as a major contributor to alarming prevalence of obesity.^4^,^5^

Attempts for ameliorating obesity have concentrated on balancing energy as core factor involved in the obesity epidemic through calorie restriction and exercise. Calorie control usually include macronutrients counting example low fat, low-carbohydrate, or high protein diet.^6^ But neither the percentage of calorie from fat, carbohydrate, or protein, nor the type of them seem to be important.^7^ But by following the above-mentioned strategy, still the role of micronutrients in energy balance and the precise metabolic pathway remains unclear,^6^ in spite of a growing body of recent evidence that has emerged in support of an “antiobesity” effect of dietary calcium and dairy products.^8^

An accumulating body of recent animal data suggests that dietary calcium and dairy products, in particular, may have a pivotal role on fat cell metabolism in a way that greater weight loss can occur despite an identical calorie intake. In this case, intracellular calcium plays a critical role in the metabolism of the adiposyte.^7^ Increasing dietary calcium without energy restriction may also facilitate a repartitioning of dietary energy from adipose tissue to lean body mass, resulting in a net reduction in fat mass and adiposity, in both mice and humans. In addition, increasing dietary calcium intake during energy restriction accelerates augmentation of body weight loss and fat loss in both mice and humans.^8^,^9^

These results are supported by epidemiological observations from NHANES III,^10^ the Quebec Family Study,^11^ the CARDIA Study^12^ and the HERITAGE Family Study.^13^

Several human studies have reported negative relations between high calcium intake and some obesity comorbidities, such as hypertension, diabetes and insulin resistance, and other studies showed an inverse association between calcium intake and body weight, body fat and the risk of becoming obese.^11^

Animal models have shown the mechanism of how low calcium intakes could affect body fat stores.^11^ The increase in circulating calcitriol (1,25-dihydroxyvitamin D), which occurs in response to low-calcium diets, stimulates adiposytes via a specific membrane vitamin D receptor to increase influx of calcium across the adipocyte membrane. As a consequence, increase in intracellular Ca^2+^ exerts a coordinated regulatory effect on adipocyte lipogenic and lipolytic system, serving to stimulate lipogenic gene expression and lipogenesis and to inhibit lipolysis, lead to increase of lipid storage and adiposity. Furthermore, calcitriol also acts using the nuclear vitamin D receptor to inhibit the expression of adipocyte uncoupling protein 2 (UCP2), thereby limiting mitochondrial fatty acid transport and oxidation.^9^,^14^

In contrast, a rise in dietary calcium induces a suppression of calcitriol levels, resulting in reduced lipogenic gene expression and increase in lipolyesis and UCP2 expression, thereby reducing lipid storage and adiposity.^10^ But, dairy sources of calcium exert significantly greater effects than calcium supplement, and this augmented effect of dairy products related to calcium supplement is likely due to the other bioactive compounds, such as angiotensin converting enzyme inhibitors, which are found in dairy, which may act synergistically with calcium to attenuate adiposity.^9^

The above-mentioned conflicting findings reveal a demand for further investigation in the context of dietary calcium intake and obesity, fat mass and insulin resistance. Thus, the present study was performed to investigate the relationship between calcium supplement intake and obesity, fat mass, insulin resistance and blood pressure, secondary to energy-restriction diets, producing an energy deficit of 500 kcal/d.

## Methods

### 

#### Study Design

This study was designed as a randomized placebo-controlled, double-blind trial to determine whether calcium supplementation accompanied with calcium carbonate will accelerate weight and fat loss and decrease the insulin and glucose concentrations and blood pressure induced by caloric restriction in 40 obese adults.

#### Subjects

Subjects were recruited to participate by placing advertisements in the Iran University of Medical Science.

One hundred overweight and obese adults were initially enrolled to participate. Of whom, 53 did not meet the inclusion criteria. The entry criteria were as followed: being aged between 20 and 60 years, BMI > 25 kg/m^2^, and an agreement to follow the study diet and calcium supplement or placebo intake as prescribed. Subjects were ineligible if they had a history of any of the following problems: endocrine disease except obesity, psychiatric problems, hepatic and renal disease, nephrolitiaze, diabetes, lactose intolerance, malignancy, rheumatic arthritis, respiratory problems, hypertension, cerebral vascular attack, or suffered any form of malabsorption syndrome, hypo and hypercalcemia, coronary heart disease, hyper and hypothyroidism, and gastrointestinal disorders. The women who were pregnant or lactating were unable to participate in the study. Finally, subjects were excluded from participation if they were taking calcium supplements in the previous 6 months, used corticosteroid and anticonvulsant or lipid-lowering, oral contraceptive drugs, antiacids, alcohol and drugs. Patients were excluded from participation if they utilized obesity pharmacotherapeutic agents and/or herbal preparations intended for any trial weight change. Of the 100 persons who enrolled, 47 subjects were eligible for the study and 7 of them were dropped; three noncompleters reported not being able to follow the diet and four subjects had medical reasons. Forty people were observed to complete the study. The study protocol was approved by the ethics committee of the Iran University of Medical Sciences.

Written consents were obtained from all subjects after they had been informed about the exact nature of the study, including the purpose, the course and the potential risk of the study.

#### Diets

Subjects were provided with individual assessments of dietary intake by 24 hour recalls, food frequency questionnaires, also they were provided with individual instruction and counseling by a registered dietitian.^15^ A diet was prescribed for every person, for 24 weeks. Basal energy expenditure based on the Harris-Benedict equation was used to calculate basal metabolic rate, which was then adjusted for activity level; estimated to be 1.4-1.5 × BEE for those who engaged in mild daily activities and 1.6-1.7 × BEE for those engaged in strenuous daily activities.^14^,^16^ Based on this initial estimate of caloric needs, diet and a food exchange was prescribed in order to produce a caloric deficit of ~500 kcal/d (diets were individualized to achieve a 500 kcal/person/day deficit). The diets were constructed to provide comparable levels of macronutrients (fat: 27.5%, carbohydrates: 52.5%, proteins: 20% of total kilocalories).

Physical activities, exercise, and tobacco and caffeine use were assessed by using questionnaires and maintained at a constant level (individualized for each person based on baseline assessment) throughout the study. After completion of baseline testing, participants were maintained on a balanced deficit diet (-500 kcal/d), matching their BMI, sex and age and were divided randomly assigned into the calcium and placebo groups for 24 weeks.

Subjects received 1 g of elemental calcium daily as the carbonate or identical placebo which was produced from lactose, starch, polyvinyl pyrolidone, and magnesium estearat.

They were asked to take two tablets (each containing 500 mg elemental calcium) in the evening with dinner.

Compliance was assessed by interviewing subjects on a weekly basis, monthly FFQ, 24 hour recalls, tablet counts and a dietitian discussed problems with the subjects to enhance adherence of the diet.

#### Anthropometric Measurement

Body weight and height, waist and hip circumferences, fat and lean mass were measured at baseline, the 12^th^ week and the 24^th^ week, by a qualified technician.

Body weight was obtained using a floor scale which was accurate to 0.01 kg, with subjects wearing light indoor clothes with no shoes, outerwear or accessories.

Height was obtained with a wall-mounted stadiometer which was accurate to 0.5 cm with subjects without shoes.

Body Mass Index (BMI) was calculated via the standard equation (kilogram per meter squared).

Waist circumference was measured in the standing position, with measurements obtained midway between the lateral lower rib margin and the iliac crest.

Hip circumference was measured at the widest point of the hip/buttocks area with the tape measure parallel to the floor.

Waist to stature ratio (WSR) and waist to hip ratio (WHR) were also calculated.

Total fat and lean mass and lean and fat mass percentages were assessed using bio electrical impedance analysis (BIA) (BODYSTAT Quad SCAN 4000-England); the instrument was calibrated. Subjects had not eaten or drunken 4-5 hours prior to measurements and also had no exercise 12 hours prior and no alcohol or caffeine consumption 24 hours prior to the test. Subjects were lying in the supine position, and it was ensured that at least 4-5 minutes has elapsed before starting the measurement.

Blood pressure measurement was taken, with the subject stated in an upright position in a chair for at least 5 minutes with the arm supported at heart level. Blood pressure was measured with calibrated sphygmomanometer on the same arm for every measurement.

#### Glucose and Insulin Measurement

Serum glucose and insulin were determined from a 6 cc blood sample, collected after the subjects had fasted overnight for at least 12 hours at baseline, at the 12^th^ week and at the 24^th^ week.

The blood samples after collection, were immediately centrifuged, and the serum was stored at -8°C.^17^

Plasma glucose was determined using a glucose oxidase method by the use of commercial kit (Parsazmun-Iran). Insulin concentration was assessed by a commercially available radioimmunoassay (IRMA) kit (Immunotech, A Beckman Coulter Company, Prague).

Insulin resistance and sensitivity were calculated with using the Homeostasis Model Assessment of Insulin Resistance (HOMA-IR) and Quantitative Insulin Sensitivity Check Index (QUICKI).

#### Nutrient Intake

Daily energy, macronutrient and micronutrient intakes were assessed for all subjects by using one per day (24 hour) recall and food frequency questionnaires handed out per assessment period (baseline, at the 12^th^ week and at the 24^th^ week).

#### Statistical Methods

A General Linear Model (GLM) procedure of repeated measurements was used for the effect of the supplementations. Values were considered significant at p < 0.015.

All data was assessed for normality of distribution before statistical analysis via Kolmogrov-Smirnov Test. Mann-Whitny nonparametric test was used for comparing means of the variables that hadn’t normal distribution, and Wilcoxon Signed Rank Test was used for comparing differences between the before and other stages of supplementations within any group. The parametric test was used for those variables which were normally distributed. The Student’s t test was used for comparing means of the variables between groups, before and after consuming supplementation and paired t test was used for comparing means of the variables within a group before and after supplementation.

Differences were considered significant at p < 0.05.

All tests were two-tailed and expressed as mean ± SD.

Only subjects who completed the entire study (n = 40) in which they were enrolled were included in the data analysis.

Information about nutrient intake which were gathered by the 24 hour recalls and by FFQ were subsequently coded, and the energy, macronutrient, and micronutrient contents of the diets were calculated by Food Processor Nutrition System vs. □□ Software.

Data was analyzed using the Statistical Package for the Social Sciences (SPSS) for Windows Version 10 (SPSS Inc., Chicago, IL).

## Results

After baseline testing, 47 participants were randomly assigned for the study, and 40 completed the 6-month protocol (85%).

There were no significant differences in any of the baseline parameters between the two groups, which made them comparable in all variables at baseline. The participants’ characteristics of the study at baseline are shown in table 1. As expected from the experimental design, all subjects lost body weight (mean ± SD) by 2.65 ± 0.13 kg due to the daily energy deficit at the 12^th^ week, but decreased 1.4 ± 0.3 kg in the calcium group at the 24^th^ week and the decrease was 1.55 ± 0.51 kg and 1.5 ± 0.44 kg in those taking the placebo at the 12^th^ week and 24^th^ week, respectively. Each of these changes were significant within the two groups (p = 0.000) but not between the group.

**Table 1:** Baseline characteristics of the subjects

Variable	Calcium group (n = 20)	Placebo group (n = 20)	P value
Age (year) *	36.6 ± 7.8	36.6 ± 8.0	1.0
Sex (male, female) **	4, 16	2, 18	0.661

All values are mean ± SD.

* There were no significant differences between two groups at baseline (Independent t- test)

** There were no significant differences between two groups at baseline(χ^2^).

The amount of the lean and fat mass showed a non significant difference between the two groups. But the lean mass showed a non significant increase in the calcium group at the 12^th^ week compared to baseline and in placebo group at the 24^th^ week compared to the 12^th^ week.

The insulin concentration showed no significant difference between decreases in both groups. But in the calcium group a significant decrease was observed at the 12^th^ week compared to the initial values (p < 0.05).

The diastolic blood pressure did not have a significant difference between the two groups, but a significant decrease was observed at the 24^th^ week compared to the 12^th^ week in both groups (p = 0.013-0.009). (Table 2)

**Table 2:** Effects of calcium supplementation on variables

Variable	Calcium group (n = 20)			Placebo group (n = 20)			P value
		
	Baseline	12^th^ week	24^th^ week	Baseline	12^th^ week	24^th^ week	
Weight (kg)	77.65 ± 16.87	75.00 ± 16.74	73.60 ± 17.04	76.25 ± 8.23	74.70 ± 8.74	73.20 ± 9.18	0.870
WC (cm)	89.40 ± 11.02	86.55 ± 10.97	85.10 ± 11.12	87.65 ± 7.74	85.20 ± 7.57	83.90 ± 7.58	0.633
HC (cm)	107.85 ± 8.23	105.70 ± 8.10	104.15 ± 8.30	108.65 ± 5.74	106.75 ± 6.35	105.20 ± 6.18	0.672
Glucose (mg/dl)	85.40 ± 4.84	75.55 ± 7.85	71.20 ± 8.53	86.60 ± 7.65	77.60 ± 7.55	73.55 ± 8.36	0.364
Insulin (mIU/ml)	6.17 ± 3.24	2.62 ± 3.83	6.88 ± 3.29	4.86 ± 3.42	4.14 ± 2.84	5.15 ± 3.87	0.510
Fatmass (kg)	26.98 ± 7.36	24.40 ± 6.48	23.66 ± 7.12	26.86 ± 6.27	25.47 ± 6.33	24.56 ± 7.06	0.773
Leanmass (kg)	50.67 ± 13.39	50.98 ± 13.16	50.18 ± 13.47	49.38 ± 6.09	49.22 ± 6.47	48.69 ± 6.04	0.647
BP (systol) (mmHg)	11.25 ± 0.96	11.35 ± 0.58	11.0 ± 0.85	11.32 ± 1.07	11.05 ± 0.82	10.58 ± 0.82	0.540
BP (diastol) (mmHg)	7.50 ± 0.76	7.75 ± 0.91	7.25 ± 0.85	7.62 ± 0.88	7.60 ± 0.82	7.35 ± 0.67	0.900

All values are mean ± SD.

There were no significant differences between groups in 3 stages of the study (general linear model).

No significant differences were observed regarding ratios of BMI, WHR, WSR and HOMA-IR and QUICKI indices. (Table 3)

**Table 3:** Effects of calcium supplementation on anthropometric and insulin resistance and sensitivity indices

Variable	Calcium group (n = 20)			Placebo group (n = 20)			P value
		
	Baseline	12^th^ week	24^th^ week	Baseline	12^th^ week	24^th^ week	
BMI	29.74 ± 3.88	28.69 ± 3.70	28.13 ± 3.86	29.77 ± 3.27	29.17 ± 3.43	28.85 ± 3.59	0.799
WHR	0.83 ± 5.67	0.82 ± 6.03	0.81 ± 6.0	0.81 ± 4.80	0.80 ± 4.76	0.80 ± 4.73	0.247
WSR	55.49 ± 4.81	53.69 ± 4.54	52.79 ± 4.73	54.79 ± 4.93	53.25 ± 4.83	52.44 ± 4.87	0.744
HOMA-IR	23.52 ± 12.77	9.15 ± 13.37	22.12 ± 11.67	18.74 ± 13.01	14.46 ± 10.40	16.31 ± 11.39	0.516
QUICKI	1.19 ± 0.45	0.34 ± 0.91	1.32 ± 0.20	1.13 ± 0.24	0.92 ± 0.59	1.09 ± 0.46	0.396

All values are mean ± SD.

There were no significant differences between groups in 3 stages of the study (general linear model).

BMI = Body mass index; WHR = Waist to hip ratio; WSR = Waist to stature ratio; HOMA-IR = Homeostasis model assessment of insulin resistance; QUICKI = Quantitative insulin sensitivity check index

Diet data for the 40 subjects who completed the study are given in table 4. There was no significant difference in the calorie and the other macro and micronutrient intakes during the study, between the two groups, at baseline, at the 12^th^ week and at the 24^th^ week.

**Table 4:** Diet characteristics of samples

Nutrient	Calcium			Placebo			P value
		
	Baseline	12 week	24 week	Baseline	12 week	24 week	
Energy (kcal/d)	1898.10 ± 1160.56	1617.75 ± 730.90	1329.05 ± 488.90	1146.15 ± 420.29	1151.00 ± 457.40	1130.25 ± 436.10	> 0.05
Protein (g/d)	57.11 ± 32.76	62.24 ± 38.30	50.23 ± 24.40	48.80 ± 25.72	42.68 ± 19.03	52.60 ± 29.22	> 0.05
Carbohydrate (g/d)	235.86 ± 124.28	173.38 ± 86.40	144.25 ± 58.61	143.40 ± 45.45	143.46 ± 64.55	147.82 ± 42.60	> 0.05
Total fat (g/d)	84.56 ± 73.69	76.54 ± 42.73	58.75 ± 33.80	47.91 ± 25.07	46.96 ± 18.37	50.59 ± 31.98	> 0.05
Saturated fatty acid (g/d)	20.54 ± 16.72	19.62 ± 10.31	50.19 ± 8.65	50.18 ± 150.49	14.19 ± 6.14	15.67 ± 10.24	> 0.05
Mono unsaturated fatty acid (g/d)	25.74 ± 22.79	22.35 ± 11.68	25.95 ± 14.42	23.52 ± 10.35	16.39 ± 9.13	17.36 ± 7.46	> 0.05
Poly unsaturated fatty acid (g/d)	33.25 ± 40.14	40.29 ± 54.15	11.74 ± 10.55	12.99 ± 10.45	12.54 ± 6.22	15.18 ± 23.38	> 0.05
Colestrole (g/d)	180.69 ± 187.32	227.38 ± 196.22	241.71 ± 198.37	159.85 ± 128.12	195.21 ± 197.72	125.30 ± 82.11	> 0.05
Zinc (mg/d)	8.56 ± 5.62	7.95 ± 5.17	7.22 ± 4.37	6.91 ± 3.50	6.26 ± 3.27	7.76 ± 3.19	> 0.05
Iron (mg/d)	13.34 ± 6.83	11.02 ± 4.97	10.61 ± 4.55	8.54 ± 2.50	9.29 ± 4.29	10.96 ± 5.70	> 0.05
Calcium intake (mg/d)	396.24 ± 479.50	394.96 ± 695.10	308.73 ± 620.70	416.19 ± 588.21	257.24 ± 547.25	581.61 ± 806.15	> 0.05

Values are mean ± SD.

There were no significant differences between groups in 3 stage of study (mann-whitny, independent t test).

There were no significance differences in the physical activities, exercise, and tobacco and caffeine use between the two groups.

## Discussion

While several longitudinal and cross-sectional studies have shown a strong inverse relation between calcium intake and body weight^6^ and body fat mass, some others have detected some evidences for affected dairy products or calcium supplementation in reducing body weight or fat mass.^17^,^18^ Because of the conflicting data, limited longitudinal clinical studies, and the increase in obesity, there is a critical need for better understanding of the effect of calcium intake on the regulation of body weight. This research is a contribution to the above-mentioned studies. No significant difference was found in weight, fat and lean mass or significant decrease in blood pressure, insulin and glucose concentrations after 6-month calcium supplementation plus calorie restricted diet between the two groups who participated in the present study.

Reid et al^19^ who performed a double-blind, randomized, controlled trial about calcium supplementation in 1471 normal post menopausal women at 30 months, found no significant change in their body weight. They also observed hypotensive effect was small and transient in most of the women. This study was very large and had a long duration, in which randomized, controlled trial had been done for the question. It had adequate power to detect a biologically significant effect on weight, but the results were the same as the present study, while their subjects had normal weight. In another randomized controlled trial Jensen et al^20^ reported that a 1 g/d calcium supplementation made no difference to weight change over a 3-month period in 62 obese women given low calorie diets, which supports the present finding, although duration of their study was shorter than the present study.

Bowen et al^21^ in a randomized, parallel study (12 weeks of energy restriction,4 weeks of energy balance) in 50 healthy overweight and obese, observed that increased dietary calcium/dairy foods in an energy-restricted, high protein diet does not affect weight loss or body composition. Also Gunther et al^17^ in a randomized, 1-year intervention for dairy calcium in 135 young healthy normal weight women with intake of dietary calcium < 800 mg/d and intake energy of 2200 kcal/d reported that an increased intake of dairy products does not alter body weight or fat mass.

In a randomized trial, Thompson et al^7^ compared a moderate-calcium diet with a high-calcium diet in a 48-week period, in 72 subjects, reported fasting glucose and insulin improved significantly, but there were no significant differences between the experimental diet and the controlled diet. This study confirms the results of the present study.

In most of the trials, subjects were not calorie restricted; however, two of them, which considered calcium supplementation vs. placebo in conjunction with a calorie restricted diet, showed no difference in weight loss.^20^,^22^ Jensen et al^20^ evaluated 52 women for 3 months comparing 800 mg in the control group and 1800 mg in the treatment group, weight loss was 6% in both groups. Another study^22^ combined data from three randomized double-blind trials and reported 1000 mg of calcium supplementation had no effect on weight loss in 100 obese women over 6 months.

Phillips et al^23^ also showed no relation between calcium intake and body composition in a non-obese adolescent population studied longitudinally over 3 years.

Barr^24^ through a review study reported that on the data available from randomized trials about dairy products or calcium supplementation, provided little support for an effect in reducing body weight or fat mass. But the reviewed studies were not specifically designed to detect the impact of calcium on body weight.

Through two studies Zemel et al^8^,^25^ found a significant reduction in BMI and waist circumference. In one of them which had a placebo controlled design comparing a high dairy diet vs. both low dairy and calcium supplemented diet, they found a significant decrease in plasma insulin^8^ in obese individuals who were on a diet rich in dairy food during low calorie diet; whereas in the second one they compared a yogurt-rich diet with a low dairy diet.^25^ In contrast, two other intervention studies involving calorie restriction^6^,^7^ did not find any significant additional decrease in body weight during a high dairy food diet in comparison to a diet low in dairy products. In these studies, which was carried out on obese subjects, those documenting an inverse association between dairy-foods and body weight lasted 24^8^ and 12^25^ weeks, whereas those that did not report any link lasted 48^7^ and 52^6^ weeks. In contrast to the present findings, these studies suggest that diet rich dairy confers protection against loss of lean body mass during energy restriction.^6^–^8^,^25^ But the results of the present study did not show this.

In one study Eagan et al^26^ determined the impact of the 18-month dairy intervention and observed a negative change in body fat mass. They found increased dietary calcium intakes through dairy products may prevent fat mass accumulation in young, healthy, normal weight women.

Jacqmain et al^11^ performed a study on 470 adults and they reported that a potential effect of calcium on body weight and fat mass was evident in the subjects, mainly in women. Their findings support Zemel et al^10^ research result, which revealed body weight, BMI, per centage of body fat, fat mass, waist circumference and abdominal adipose tissue were all significantly greater in women with a low calcium intake.

A number of epidemiologic studies have proposed a profit of dietary calcium on weight. An analysis of the National Health and Nutrition Examination Survey III dataset reported that weight was inversely related to calcium intake.^10^ After controlling for age, energy intake, and socioeconomic status, The Quebec Family Study^11^ found a low calcium intake (< 600 mg calcium daily) was predictor for the risk of becoming obese, having higher waist circumference and having more body fat in women, whereas difference in men was not significant. However, Kamycheva et al study^27^ was observed a positive association between calcium intake and BMI in men but not in women. Unlike the above cross-sectional studies, Bostick et al Study^28^ reported no relationship between calcium intake and BMI or waist to hip ratio.

In CARDIA study Pereira et al^12^ observed an inverse association between frequency of dairy intake and the development of obesity, abnormal glucose homeostasis and elevated blood pressure in young overweight black men and women. But this study was an observational by nature and a longitudinal one. Azadbakht et al^29^ in another observational study on 827 adults aged from 18 to 74 years observed that dairy consumption was inversely associated with the risk of having metabolic syndrome, which seems somewhat attributed to the calcium. Mennen et al^30^ in a study which was performed by FFQ on 2537 women and 2439 men concluded that high consumption of bread or dairy products may be related to the risk of metabolic syndrome in men. But because they did not have related data on all major types of the food consumed by the subjects it cannot be concluded that a high consumption of bread or dairy products is a marker of consumption of something else.

Devies et al^31^ and Heaney et al^32^,^33^ have reevaluated data from nine studies, including three controlled trials and six observational studies, about calcium intake in which body weight could be assessed as a secondary outcome. They found, significant negative associations between calcium intake and body weight, body fat and/or weight gain.

In addition to the clinical and epidemiological data, data from animal studies also suggest an effect of calcium on weight. Papakonstantinou et al^34^ reported that rats fed a high calcium diet which was also supplemented with milk protein had 29% less carcass fat. This was contradicted by similar studies in lean and obese mice and rats reported by Zhang and Tordoff.^35^ They could not confirm the positive effects of calcium on body weight or carcass fat content in obese and normal weight rats. This explanation might also apply to other animal studies. Zemel et al^10^ have reported in a study on mice overexpressing the agouti gene. They documented that dietary calcium supplementation produced a dose-related diminution in weight gain and fat mass.

A relation between calcium intake and blood pressure has been reported from observational studies in a variety of population,^36^–^38^ typically showing a fall in both systolic and diastolic blood pressure of about 0.4 mm Hg for increase of 100 mg in daily calcium intake.^39^ All studies that have found a beneficial effect of calcium on blood pressure have been less than 6 months in duration, which suggests that the hypotensive effect of calcium is real, but transient. Griffith et al,^40^ who performed a meta-analysis of the effect of calcium on blood pressure, found a small statistically significant effect of calcium on lowering systolic blood pressure independent of baseline blood pressure, and a small trend toward reduction in diastolic blood pressure that did not reach statistical significance. Another important finding has to do with their prior hypothesis is that dietary and nondietary intervention may differ in their effects on blood pressure reduction.

One of the possible physiological mechanisms for the effect of calcium intake on fat mass is by the calcitrophic hormones, PTH and 1,25 (OH) 2D. Both PTH and 1,25 (OH) 2D have been proposed to decrease fat breakdown and to increase fat synthesis, potentially through increases in the amount of intracellular calcium in adipocytes.^10^ Dairy sources of calcium exerted significantly greater antiobesity effects when compared with a supplemental calcium carbonate in each of these studies, consistent with the clinical results presented here. Although the additional factors in the dairy responsible for this effect have not been identified yet, but it has been recently suggested that milk is recognized as a rich source of bioactive compounds^41^ that may also act independently or synergistically with the calcium to suppression of 1,25-dihydroxyvitamin D3 to favorably affect nutrient portioning, metabolic efficiency, and fat loss.^42^

The above-mentioned discrepancies between different studies may possibly be related to the different amounts of calcium supplement prescribed in different groups, participated in the reviewed studies in this paper, or can be related to the period of supplementation, or both, which all can be evaluated as important factors. For example, discrepancy between many of these studies and the present one about change in body composition is related to the different tools which were used for the assessments. In the present study, body composition assessed by bioelectrical impedance, while others used DEXA.

On the other hand there should be a possibility of a threshold effect that is, if the calcium intake is < 600 mg daily [such a threshold is suggested by the Quebec Family Study^11^], then weight loss might be diminished.^7^ It is possible that calcium enhances weight and fat loss best when individuals have very low baseline intakes (< 500 mg/d).^28^ However, Shapses et al found no effect of calcium supplement on weight and fat losses in two groups of women regardless of baseline calcium intake, with some subjects consuming ~700 mg/d of calcium at the baseline, and others using up to ~1000 mg/d, which suggests, there is no threshold effect for usual calcium intake.^22^

It is not clear how these factors contribute to the different studies, which lead to different results. Following this question is suggested to be considered in further research plans.

## Conclusions

In conclusion the major present findings reveal that a calorie restricted diet with intake of 1 g/d calcium supplementation as the carbonate, for 24 weeks does not significantly lead to alteration in body weight, fat and lean mass. It was found that blood pressure, glucose and insulin concentrations and HOMA-IR and QUICKI between two groups were not significantly different beyond what can be achieved in a behavioral weight loss intervention.

This was not a short term trial and the length of the study was logical and suitable; however, more longitudinal studies suggested to be conducted in future. Different studies had shown the importance of longitudinal clinical trials.^43^

The strengths of this study include: a six month follow up, a low drop-out rate (15%), and a randomized double-blind controlled trial design. Also comparing with the other studies, subjects in the present study were on a calorie restricted approach. The weakness of this study was the lack of measurement of skinfold thickness.

But more controlled clinical trial with sufficient numbers of participants are needed to establish whether there is a casual association between calcium intake and body weight and they should take possible confounders into consideration, such as gender, ethnicities, age, degree of obesity and habitual intake of calcium or dairy products.

There is consistent evidence from randomized controlled trials that calcium supplementation slows postmenopausal bone loss,^44^ and there is also some evidence that it prevents fractures in postmenopausal women.^45^,^46^ Other benefits from the use of calcium supplements have been suggested, including effects on colon cancer,^47^ and lipoprotein concentration.^48^ There is also observational evidence that calcium intake is inversely associated with cardiovascular disease.^27^ So, if established that calcium intake causes a decrease in the BMI and body weight, fat mass and HOMA-IR and blood pressure, the use of the calcium supplement or dairy intake leads to prevention of several diseases.
